# Multi-Dimensional Health Assessment Questionnaire in China: Reliability, Validity and Clinical Value in Patients with Rheumatoid Arthritis

**DOI:** 10.1371/journal.pone.0097952

**Published:** 2014-05-21

**Authors:** Yang Song, Li-an Zhu, Su-li Wang, Lin Leng, Richard Bucala, Liang-Jing Lu

**Affiliations:** 1 Department of Rheumatology, Ren Ji Hospital, School of Medicine, Shanghai Jiao Tong University, Shanghai, China; 2 Department of Medicine, Section of Rheumatology, Yale University School of Medicine, New Haven, Connecticut, United States of America; Center for Rheumatic Diseases, India

## Abstract

**Objective:**

To evaluate the psychometric properties and clinical utility of Chinese Multidimensional Health Assessment Questionnaire (MDHAQ-C) in patients with rheumatoid arthritis (RA) in China.

**Methods:**

162 RA patients were recruited in the evaluation process. The reliability of the questionnaire was tested by internal consistency and item analysis. Convergent validity was assessed by correlations of MDHAQ-C with Health Assessment Questionnaire (HAQ), the 36-item Short-Form Health Survey (SF-36) and the Hospital anxiety and depression scales (HAD). Discriminant validity was tested in groups of patients with varied disease activities and functional classes. To evaluate the clinical values, correlations were calculated between MDHAQ-C and indices of clinical relevance and disease activity. Agreement with the Disease Activity Score (DAS28) and Clinical Disease Activity Index (CDAI) was estimated.

**Results:**

The Cronbach's alpha was 0.944 in the Function scale (FN) and 0.768 in the scale of psychological status (PS). The item analysis indicated all the items of FN and PS are correlated at an acceptable level. MDHAQ-C correlated with the questionnaires significantly in most scales and scores of scales differed significantly in groups of different disease activity and functional status. MDHAQ-C has moderate to high correlation with most clinical indices and high correlation with a spearman coefficient of 0.701 for DAS 28 and 0.843 for CDAI. The overall agreement of categories was satisfying.

**Conclusion:**

MDHAQ-C is a reliable, valid instrument for functional measurement and a feasible, informative quantitative index for busy clinical settings in Chinese RA patients.

## Introduction

Rheumatoid arthritis (RA) is one of the few diseases where subjective patient and physician measures are the best known predictors of treatment response and future health outcomes [1]. The treatment of RA has been improved greatly by current regimens of disease modifying drugs and biologic agents. From the patients' perspective, however, many deleterious disease consequences still exist, including persistent pain, functional disability, fatigue, and depression that may be affected by health beliefs and underlying psychological problems [2]. Quantitative measurement of such information is therefore critical. Patient self-report questionnaires have been reported to be the most cost-effective in the documentation of the effectiveness of rheumatology care [3], [4], and standardized patient questionnaire measures, rather than laboratory tests or radiographs, are the most significant quantitative predictors of severe outcomes of rheumatoid arthritis (RA), including work disability [5]–[7] and mortality [8], [9].

The heath assessment questionnaire (HAQ) is widely used throughout the world to assess functional status in rheumatoid arthritis and a wide variety of rheumatic diseases. Developed in 1978, the HAQ remains the gold standard for measuring functional status in RA [10]. However, its length and relatively complex scoring could make clinical use difficult. Accordingly, several revisions have been made over past several years. The multidimensional health assessment questionnaire (MDHAQ) is the latest version of such revisions with decreased patient and provider time requirement [1]. Meanwhile, it has a broader perspective and better coverage of the scales in the International Classification of Functioning, Disability and Health (ICF) [11], [12] The MDHAQ also includes the routine assessment of patient index data 3 (RAPID3), an index that includes three of the patient-reported American College of Rheumatology (ACR) core data set measures for RA: physical function, pain, and patient global estimate of status [13]. RAPID3 has been reported to give similar data as DAS28 and CDAI for distinguishing active versus placebo treatments in clinical trials [14] and is calculated much more easily and quickly than HAQ, DAS28 and CDAI [15].

In this study,the original English version of MDHAQ was translated into Chinese with an cross-cultural adaptation process, and an assessment of psychometric properties and values was performed in Chinese patients with RA.

## Patients and Methods

### Ethics Statement

This study was approved by the institutional review board of Shanghai Jiao Tong University and Ethics Committee of Renji hospital. They specifically approved that written informed consent was not required because data were going to be analysed anonymously. Following feedback from participants in the pretest procedure, all participants granted oral consent to participate after receiving comprehensive information about the study. Oral consent was documented by interviewers at the beginning of the interview.

### Patients

One hundred and sixty-two consecutive RA patients were recruited into the study between March 2013 and November 2013 from Ren Ji Hospital, School of Medicine, Shanghai Jiao Tong University. Each patient had to fulfill the following criteria: (a) met the ACR 1987 revised criteria for the classification of RA [16] (b) was at least 18 years of age. The patients were excluded if they had chronic disabling disease other than RA or cognitive impairment. Demographic characteristics were recorded including age, sex, marital status, education, and disease duration.

### MDHAQ

The MDHAQ is a 2-page version questionnaire which comprises 10 questions regarding physical function(FN), psychological status(PS), pain(PN), Rheumatoid Arthritis Disease Activity Index (RADAI) self report joint count (JTCT),global health status(PTGL), fatigue(FT), symptom checklist review of systems (ROS),morning stiffness(AM), exercise habits(EX), and change in status over the last week(CHG) as well as a recent medical history [17], [18]. The FN includes 10 items, numbered Question1.a–j, on activities of daily living scaled in a Likert format (from 0  =  without any difficulty to 3  =  unable to do, total score ranges from 0 to 10). The sum of the raw score is divided by three to give a score between 0 and 10.Three items, numbered Question1, k–m, constitute the psychological status section scored 0–9.9(scored 0  =  “without any difficulty”, 1.1  =  “with some difficulty”, 2.2 = “with much difficulty”, and 3.3  =  “unable to do”). PN, PTGL, and FT were assessed by three VASs presented as 21 circles, with an arithmetic scale of 0–10 in 0.5 unit increments in Question 2, Question 4 and Question 9. The JTCT (Question 3) includes 8 joints or joint groups, scored 0, 1, 2 or 3 by the patient. ROS (Question 5) is a quantitative review of symptoms the patient experienced over the last month, scoring of which is the number of checked boxes. AM (Question 6) is scored in minutes with maximum 300 minutes. CHG, as the 7th question, is scored 1–5(1  =  Much better, 2  =  Better, 3  =  Same, 4  =  Worse,5  =  Much worse). EX (question 8) cares about the frequency of aerobical exercises for at least 30 minutes, with a scoring instrument of 3 = 3 or more times a week, 2 = 1–2 times per week,1  =  1–2 times per month, 0  =  Do not exercise regularly,9  =  cannot exercise due to disability/handicap. And recent medical history (Question 10) is the only one that does not scored quantitatively in the whole questionnaire. RAPID3, on a 0–30 scoring scale, can be divided into four categories: high severity >12, moderate severity  =  6.1–12, low severity  =  3.1–6, and remission <  = 3.

### Translation and cross-cultural adaptation

The procedure of translation and cross-cultural adaptation was performed following the guidelines by Guillemin et al. [3], [19] The original MDHAQ was first translated into Chinese independently by 2 translators who were aware of the objectives of the questionnaire. The translated versions were then back translated into English by another two translators blinded to the intent and the concepts of the questionnaire. A multidisciplinary consensus committee was held to produce a synthesized version based on the translations and back-translations. This version was applied to 10 randomly selected outpatients with RA in the following pre-test. On the basis of the feedback and a discussion within the commitment, several wording revisions were introduced to suit the Chinese culture. “Walk two miles or three kilometers” was modified to “Walk six Li or three kilometers?”because most Chinese people use” Li”, rather than” mile” as a commonly used unit of length in everyday life.” How much of a problem has unusual fatigue or tiredness been for you” was changed to “How much unusual fatigue or tiredness have you had?” All the modifications were conceptual equivalence of the source by the commitment. An additional 10 outpatients completed this modified version and no further suggestions were feed backed. This modified instrument, referred to as the MDHAQ-China (MDHAQ-C), was administered to recruited RA patients in this study.

### Evaluation methods

Patients who completed MDHAQ-C were also asked to complete the Chinese HAQ [20], the Chinese version of the 36-item Short-Form Health Survey (SF-36) [21], and the Chinese version of the Hospital anxiety and depression scales(HAD) [22] at the same time. Disease Characteristics were estimated by three different methods: laboratory tests, clinical estimates by physicians, and patient self-estimate scales. The laboratory tests included erythrocyte sedimentation rate (ESR), C reactive protein (CRP) and rheumatoid factors (RF). A physician clinically evaluated the patient by assessments of swollen joint counts (STC) and tender joint counts (TJC) and by the physician overall assessment using a visual analog scale (VAS) and ACR functional classification [23]. Patient self-estimate scales were two VASs in a 100 mm line to assess of pain and global status completed by patients independently. Reliability was tested by internal consistency with a following item analysis procedure. Convergent validity was tested by examining the correlation of MDHAQ with HAQ, SF-36 and HAD using spearman's method. The correlations between MDHAQ and the SF-36 were hypothesized to be negative and those with HAQ and HAD were expected to be positive, with all of these correlations significant. Highly strong correlation was set as the spearman's coefficient >0.70, strong as 0.5–0.7, moderate as 0.30–0.5, and weak as <0.3. Discirminant validity was estimated in patients distributed into two different groups: patients that were active versus inactive (remission) in disease activity groups by the cutoff value of 2.6 according to DAS28, and patients with disability versus without disability groups by the cutoff value of 1 in functional class. Clinical values were assessed by comparing RAPID3 and the components with disease characteristics and agreement of RAPID3 with Disease Activity core-28(DAS28) [24] and with Clinical Disease Activity Index(CDAI) [25] Categories for disease activities were also assessed according to the statistic methods by Pincus T [13] which will be shown in detail in the following statistical analyses.

### Statistical Analyses

Test-retest was evaluated by intraclass correlation coefficient (ICC) or kappa statistics. Internal consistency was assessed using Cronbach's coefficient alpha. Item analysis was performed by item-total correlations and corrected item-total correlations. Comparisons between different groups were calculated by non-parametric test (Mann-Whitney). Spearman's rank-order correlation coefficient was used to evaluate correlation of RAPID3 and components with disease characteristics. Agreement of RAPID3 with DAS28 and CDAI was evaluated respectively by correlations using spearman's method. Cross-tabulations were computed to compare the number and proportion of patients classified in the four DAS28 and CDAI categories of high disease activity (DAS28 >5.1, CDAI >22), moderate disease activity (DAS28 = 3.21–5.1, CDAI = 10.1–22), low disease activity (DAS28 = 2.61–3.20, CDAI = 2.81–10), and remission (DAS28≤2.6, CDAI = 0–2.8) with the four proposed RAPID3 categories. The level of agreement of categories by the different scales was evaluated using kappa statistics. Statistics were considered to be significant when p value>0.05.

## Results

156 of the 162 recruited RA patients completed the questionnaires, indicating a response rate of 96.3%. All the patients were Chinese. The mean (SD) age of the patients was 46.79(12.80), ranging from 19 to 75; 87.2% of them were female and 94.2% had been married. The mean (SD) years of education was 9.32(5.62), ranging from 0 to 31 years. The mean (SD) disease duration was 6.46(7.37) years. The mean (SD) of ESR, CRP, DAS28 and CDAI was 29.6(26.5), 11.6(26.5), 4.64(6.36), and 15.08(15.61) respectively. With respect to work status, 46.8% (73) of RA patients were engaged at work, while the other 73 patients were unemployed, with 16 disabled, 39 homemakers, and 28 retired. The detailed demographic and clinical characteristics were shown in Table 1

**Table 1:** Demographic and clinical characteristics of the RA patients.

Variables	Measures
Age, years, mean(SD)	46.8 (12.8)
Sex,male/female(%)	20 (12.8)/136 (87.2)
Education,years,mean(SD)	9.3(5.6)
Marriage status, yes/no (%)	147 (94.2)/9 (5.8)
Disease duration,years,mean(SD)	6.46(7.37)
RF, positive,n(%)	104(66.7)
ESR, mm/h, mean(SD), median(IQR)	29.6(26.5), 20(11, 39)
CRP, mg/L, mean (SD), median(IQR)	11.6(26.5), 3.3(1, 12.2)
TJC, mean (SD), median(IQR)	5.6(7.3), 2(1, 7)
SJC, mean (SD), median(IQR)	2.9(4.8), 1(0, 3)
DAS28, mean(SD), median(IQR)	4.64 (6.36), 3.5(2.56, 4.73)
CDAI, mean(SD), median(IQR)	15.1(15.6), 9.5(4, 21)
HAQ, mean(SD), median(IQR)	0.51(1.39), 0.13(0, 0.5)
HAD, mean(SD), median(IQR)	8.02(6.19), 7(3, 11.8)
RAPID3, mean(SD), median(IQR)	7.94(6.52), 6.9(2.3, 12.0)
Work Status, n (%)	
Full-time	52 (33.33)
Part-time	6 (3.85)
Disabled	16(10.26)
Homemaker	39 (25)
Self-Employed	15 (9.62)
Retired	28 (17.95)

### Reliability

The internal consistency value was 0.944 in FN and the removal of each item didn't lead to a significant change in Cronbach's alpha, ranging from 0.934 to 0.943. For the PS scale, the Cronbach's alpha was .0.768 and the Cronbach's alpha rose to 0.900 when the item k was deleted while the value fell to 0.577 and 0.548 separately for item l and m (Table 2)

**Table 2:** Internal consistency and item analysis of FN and PS.

Items	Corrected Cronbach's alpha	Item-total	Corrected item-total
a.	0.936	0.670	0.800
b.	0.936	0.662	0.828
c.	0.940	0.600	0.745
d.	0.937	0.700	0.776
e.	0.935	0.725	0.815
f.	0.935	0.636	0.818
g.	0.943	0.549	0.648
h.	0.934	0.696	0.838
i.	0.940	0.738	0.743
j.	0.939	0.808	0.782
k.	0.900	0.756	0.411
l.	0.577	0.785	0.698
m.	0.548	0.801	0.733

Corrected Cronbach's alpha: the consistency coefficient of the remaining items when one item was deleted; Item-total: correlation between the item and the corresponding domains; Corrected item-total: correlation between the remaining items and the sales after one item was deleted. Statistics were significant at the level of p<0.001.

### Item analysis

The results of item analysis were displayed in Table 2. The associations between items and the scale were satisfactorily high. The item-total correlation analysis showed that all the items were correlated with FN significantly (0.549–0.808 for FN and0.756–0.801 for PS, p<0.001).The corrected item-total correlation analysis showed that the value ranged from 0.648 to o.838 for FN and 0.411 to 0.733 for PS.

### Validity

To assess the convergent validity of the MDHAQ-C, the scales of MDHAQ-C were compared to HAQ, SF-36 and HAD. As shown in Table 3, the FN had a highly strong correlation with HAQ with a coefficient of 0.859 (p<0.001) and moderate to highly strong correlation with all the scales of SF-36, ranging from 0.528 to 0.854 (p<0.001). When comparing the rest of the scales of MDHAQ-C with these criterions, most of the results were satisfying significant. Except for EX (which showed a relatively weak correlation), the remaining scales all showed moderate to strong correlation with the criterions (p<0.001) with HAQ. The results of the comparison with SF-36 was similar, with a moderate correlation (levels of significance ranging from p<0.01 to p<0.001). The MDHAQ-C also showed a significant correlation with HAD but at a relatively lower level(r = 0.379–0.564) for most scales. EX showed a significant correlation with SF (p<0.01) and VT (p<0.05), but were not significant for the other scales of SF-36 (Table 3).

**Table 3:** Convergent validity of MDHAQ-C.

			FN	PS	PN	JCTC	PTGL	FT	EX
HAQ			0.859***	0.423***	0.676***	0.622***	0.592***	0.436***	0.195*
HAD			0.397***	0.564***	0.416***	0.426***	0.379***	0.461***	0.116
SF-36		PF	−0.854***	−0.477***	−0.519***	−0.487***	−0.520***	−0.531***	−0.198
		RP	−0.705***	−0.350***	−0.515***	0.361**	−0.467***	−0.511***	−0.189
		RE	−0.546***	−0.247**	−0.485***	0.418***	−0.523***	−0.355**	−0.037
		SF	−0.537***	−0.501***	−0.472***	0.453***	−0.451***	−0.477***	−0.346**
		BP	−0.563***	−0.305**	−0.626***	0.512***	−0.541***	−0.381***	−0.115
		GH	−0.528***	−0.373***	−0.548***	0.413***	−0.525***	−0.390***	−0.171
		VT	−0.645***	−0.509***	−0.574***	0.460***	−0.511***	−0.640***	−0.256*
		MH	−0.554***	−0.523***	−0.464***	0.495***	−0.508***	−0.543***	−0.242*

PF: physical functioning, RP and RE: physical and emotional roles, SF: social functioning, BP: bodily pain, GH: general health, VT: vitality, MH: mental health. All the above belongs to the scales of SF-36.

*: p<0.05, **: p<0.01, ***: p<0.001.

Results of assessing of discriminant validity performed on the total10 scales of the MDHAQ-C are detailed in Table 4. Mean scores on the MDHAQ-C differed significantly between patients who were active in disease status and those who were in remission based on DAS28 scores in all scales except for CHG and EX. Similarly, all scales could differentiate subjects without functional disability from those with that situation judged by functional class in all scales except EX.

**Table 4:** Discriminant validity of MDHAQ-C,with functional class and disease activity being the external anchor.

	functional class		p value	DAS28		p value
	1	>1		≤2.6	>2.6	
FN	0.33(0.64)	2.63(2.19)	0.000	0.48(1.42)	1.47(0.18)	0.000
PS	0.93(1.21)	2.58(2.26)	0.000	1.04(1.45)	1.73(1.94)	0.033
PN	1.76(1.91)	5.80(2.59)	0.000	0.92(1.20)	4.16(2.94)	0.000
JCTC	3.60(5.23)	15.43(11.69)	0.000	1.90(4.79)	10.40(10.64)	0.000
PTGL	2.23(2.06)	5.28(2.15)	0.000	1.63(1.78)	4.06(2.51)	0.000
ROS	4.16(3.40)	7.98(5.42)	0.000	3.72(2.99)	6.27(4.98)	0.002
AM	19.74(55.54)	57.30(82.35)	0.000	19.87(62.80)	38.46(69.70)	0.005
CHG	2.62(0.75)	2.96(0.53)	0.047	2.73(1.56)	3.25(3.61)	0.328
FT	2.67(2.22)	5.12(2.74)	0.000	2.18(2.16)	4.01(2.73)	0.000
EX	1.62(2.16)	3.59(4.07)	0.063	1.51(1.75)	2.68(3.49)	0.466

### Clinical value

RAPID3 and the components were compared with results of the three different methods used in the assessment of disease characteristics which has been described in detail in the evaluation methods. As shown in Table 5, RAPID3 score had strong correlation with TJC, pain, global status and physician overall assessment (p<0.001), and had moderate correlation with ESR, CRP and SJC (p<0.001). The three components of RAPID3 showed similar results: strong correlation was seen between FN and physician overall assessment, PN and TJC, PN and physician overall assessment, PN and patient self-estimating pain and global status, and between PTGL and patient self-estimating pain and global status (p<0.001). For RF, the correlation was not significant for both RAPID3 and the components. The rest of the variables showed moderate correlation with each other.

**Table 5:** Correlations of RAPID3 and components with disease characteristics.

		FN	PN	PTGL	RAPID3
Lab	ESR	0.488***	0.408***	0.415***	0.461***
	CRP	0.447***	0.444***	0.389***	0.470***
	RF	−0.045	−0.007	0.011	0.011
Physician	SJC	0.557***	0.642***	0.521***	0.628***
	TJC	0.621***	0.738***	0.648***	0.731***
	VAS of overall assessment	0.717***	0.781***	0.686***	0.808***
Patient	VAS of Pain	0.673***	0.923***	0.773***	0.885***
	VAS of Global status	0.697***	0.720***	0.865***	0.837***

Lab: laboratory tests, Physician: clinical estimates by physicians, Patient: patient self-estimate scales.

***: p<0.001.

**Figure 1:**
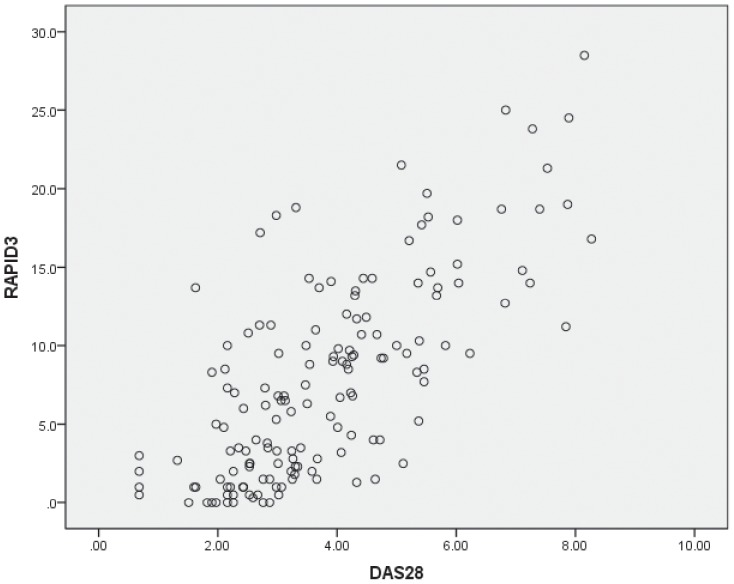
Scatter plots of correlation between RAPID3 and DAS28 in 156 patients with Rheumatoid Arthritis (spearman's method).

**Figure 2:**
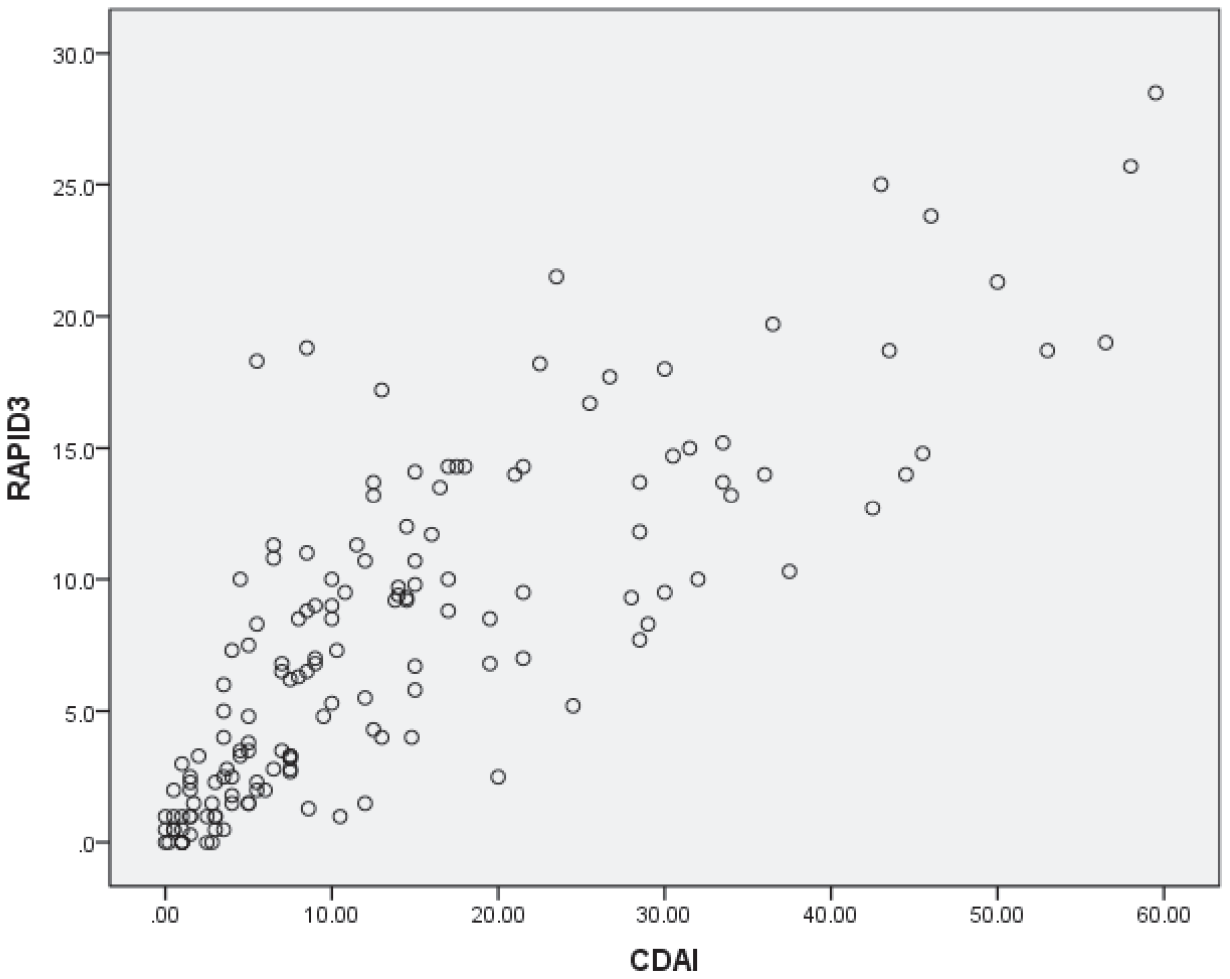
Scatter plots of correlation between RAPID3 and CDAI in 156 patients with Rheumatoid Arthritis (spearman's method).

When comparing RAPID3 with DAS28 and CDAI, Spearman's rank-order correlation coefficients were evaluated respectively. RAPID3 and DAS28 had high correlation with a coefficient of 0.701(p<0.001) (Figure 1). The kappa value was 0.467(p<0.001). Among the 156 patients, 70.6% of them who met DAS28 high activity criteria met corresponding RAPID3 criteria. The percentage was 64.8%, 33.3% and 65.8% respectively for moderate, low, and remission groups according to the category of DAS28 (Table 6). The comparison with CDAI turned out to be similar. As was shown in Figure 2, the Spearman's rank-order correlation coefficient was 0.843(p<0.001). The kappa value was 0.491(p<0.001). The percentage of patients who met high, moderate, low and remission activity criteria of RAPID3 in those who met corresponding CDAI criteria were 74.3%,52.6%,42.9% and 96.3% respectively(Table 6).

**Table 6:** The cross-tabulation of RAPID3 scores compared with DAS28 and CDAI in four categories.

	Group	RAPID3				Total
		H	M	L	R	
DAS28	H	24(70.6%)	8(23.5%)	1(2.9%)	1(2.9%)	34(21.8%)
	M	9(16.7%)	35(64.8%)	9(16.7%)	1(1.9%)	54(34.6%)
	L	2(6.7%)	9(30.0%)	10(33.3%)	9(30.0%)	30(19.2%)
	R	1(2.6%)	6(15.8%)	6(15.8%)	25(65.8%)	38(24.4%)
Total		36(23.1%)	58(37.2%)	26(16.7%)	36(23.1%)	156(100%)
CDAI	H	26(74.3%)	8(22.9%)	1(2.9%)	0	35(22.4%)
	M	10(26.3%)	20(52.6%)	5(13.2%)	3(7.9%)	38(24.4%)
	L	2(3.6%)	20(35.7%)	24(42.9%)	10(17.9%)	56(35.9%)
	R	0	0	1(3.7%)	26(96.3%)	27(17.3%)
Total		38(24.4%)	48(30.8%)	31(19.9%)	39(25.0%)	156(100%)

All percentages are row percentages, except total in rightmost column (column percentages).The kappa value was 0.467 for DAS28 and 0.491 for CDAI (p<0.001).H: high activity; M: moderate activity; L: low activity; R: remission.

## Discussion

RA is characterized by chronic destructive inflammatory polyarthropathy with a major impact on physical and psychological health. The prevalence rates range from 0.2 to 0.93% in China [26], and as Chinese people make up almost one-quarter of the world's population, the number of RA patients is quite large in China. MDHAQ is a new instrument derived from HAQ and used in the evaluation of functional status and many other aspects regarding the quality of life of RA patients. It unifies 3 important features of a rheumatologic assessment instrument: the physical and mental aspects of the patient's functioning [27], a patient oriented perspective [28], and a brief, patient friendly format [29]. The original English version has been well validated [30] but there has not been a cross-cultural version for the substantial RA patients in China. This is the first attempt to translate the MDHAQ into Chinese and evaluate its utility in assessing the health status of Chinese RA patients. In addition, this is the first time when assessment of clinical utility was added to the evaluation process of a cross-cultural adapted questionnaire.

There are many dialects that are quite different from one another such as Shanghai-ese, Cantonese and Mandarin in China although the written language is the same. Mandarin was used throughout the process and all the bilingual interpreters spoke Mandarin fluently, because Mandarin is the common language in China and almost every Chinese could communicate with each other in Mandarin no matter what his or her native dialect is. These efforts were made to ensure optimal cultural adaptation for the whole country.

The patients recruited were all with a consecutive diagnosis of RA. The Inclusion criteria were set to ensure all the patients included were adults. Since co-existing conditions are known to affect self-reported disabilities in the general population [31], careful selection and exclusion criteria are essential to exclude the confounding factor. The chronic disabling conditions excluded were those determined to be not from the disease itself, but could have an influence on daily activities, such as heart failure and diseases of respiratory system like COPD. The co-morbidity that was associated closely with RA such as interstitial lung disease was not included in the exclusion criteria to ensure the generalizability of the results. However, more work is needed in studying the excluded population of RA patients with these co-morbidities. Most patients were middle aged women who have been married and the female to male ratio was 6.8:1, which was similar with the epidemiological characteristics of RA in China [26]. As our clinical site is a major tertiary referral center for rheumatology in China, the recruited patients are geographically diverse and broadly representative of Chinese RA patients. These RA patients also represent a wide spectrum of disease and encompass a wide range of of age, education, and disease duration.

The internal consistency was good in FN and PS. However, when this assessment was conducted item by item, the results varied among the items. Item l and item m played an important role in the consistency as when the two items were deleted, the value of Cronbach's alpha decreased dramatically. Item k appeared to have a negative effect on the consistency. This result was also found in Finish [32] and Swedish studies [33]. The item-total correlations were satisfying while the corrected item- total correlation was 0.411, a value lower than the other two items, which also was consistent with the previous study. Arkela-Kautiainen believes the removal of the k item and altering the format of the item from the Likert scale to a VAS would be helpful [32]. However, when assessing if the item should be removed from an scale, the cutoff value for the criterion for corrected item-total was >0.4 according to Power [34] and Chiou [35]. The research by Ekback *et al* set the acceptable level to >0.3 [36]. In addition, the internal consistency of PS was at satisfyingly good level of 0.768 rather than a moderate consistency with a Cronbach's alpha of 0.66 in the Finish version. Thus, the removal of k item might be not appropriate in our MDHAQ-C version and the items of PS should be maintained as the original version according to our results.

The results from the analyses of convergent validity were consistent with the predicted hypotheses for most scales. Since ROS, AM and CHG were not relevant in the content with those questionnaires, this test was conducted in the remaining seven scales. The FN had strong correlation with HAQ and PF of SF-36, and a moderate to strong correlation between PS and HAD and MH of SF-36,between PTGL and GH of SF-36, and between FT and VT of SF-36, supporting good convergent validity. The other scales also correlated with HAD and SF-36 significantly except EX, which was only significantly correlated with SF, MH, and VT of SF-36. The possible reason for this effect could be that exercise is influenced by many factors such as motivation and belief [37], and a substantial of patients with RA were physically inactive [38]. This could lead to the insensitivity to change and could probably explain for the deficiency of discriminant validity of this scale in patients with different disease activities and functional status. Modifications and reformulation could be expected in our future researches. The CHG could distinguish patients without functional disability from those with disability while the mean score didn't differ significantly in active versus inactive (remission) group. As functional status was assessed by ACR functional class, of which the content was related closely with the daily activities, disability could be more easily perceived by patients than DAS 28 which contains objective results of laboratory tests. Since CHG depended totally on patients subjective feelings, that might be a possible explanation for that inconsistency. Overall, the MDHAQ-C had good discriminant validity in patients with varied disease activities and functional status.

Quantitative clinical assessment has advanced more effective treatment of RA and is associated with better outcomes than usual non-quantitative care of RA [5], [39]. A formal quantitative swollen and tender joint count and indices that include the count, such as the DAS28 and CDAI, are the most specific measures of RA activity [40]. However, these are time consuming and therefore not available at most visits of patients with a rheumatologist, particularly in busy clinical settings. RAPID3 is an index without formal joint counts that can be completed in less than 10 minutes, which is less than 10% of the time of a CDAI or DAS28 [41], [42], indicating considerable value for usual RA care in busy clinical settings.

Several different methods or indices are used commonly in clinical practice and research, including laboratory testing, and physician- or patient-based clinical assessments. As RAPID3 is a comprehensive instrument composed of three domains of MDHAQ, we compared RAPID3 and its components with methods or indices of assessing clinical relevance. This is the first time that both the RAPID3 and its components were assessed by comparison with the existed instruments, including simple ones such as TJC, SJC and others shown in Table 5, and comprehensive ones like DAS28 and CDAI. Moderate to strong correlations were seen between RAPID3 and almost all the disease characteristics, so were the three components. The correlations with RF were not significant. That was understandable because changes in titers of RF generally occurred slowly and often lagged behind other markers of RA activity, and it often occurred that RF values did not decrease along with clinical improvement in RA patients in clinical practice. RAPID3 scores correlated strongly with DAS28 and CDAI with a high coefficient of 0.701 and 0.843 respectively as shown in Figure 1 and Figure 2.When assessing the agreement of the categories of disease activity, the kappa values were acceptable at a fair to good level with 0.467 for DAS28, 0.491 for CDAI. Those results were consistent with the original research [13]. Thus, RAPID3 was as informative as other indices for disease status and provides a feasible, informative quantitative index for busy clinical settings, indicating substantial clinical utility.

## Limitations and Further Research

The patients enrolled were chosen randomly following the inclusion and exclusion criteria. Thus, the number of patients with different disease activities and functional status might not be equal with one another, which is a limitation of our research. Other limitations include sensitivity to change of MDHAQ and the modification of EX domain and further researches are needed. Also, further research could be done regarding the RAPID3 and other disease variables. Our research was performed in the setting of tertiary referral center, one of the most reputable centers for rheumatology in China. Thus our patients are from around the country and geographically diverse to make certain the results were representative for all the RA patients. However, it still remains open to question whether a community setting would make a difference for MFHAQ-C, as the lack of proof regarding scorer reliability of MDHAQ. This could be figured out in further researches.

## Conclusion

The MDHAQ-C has good reliability, validity and substantial clinical utility in Chinese RA patients. Further work should be carried out and more evidences are needed for the modification of the EX scale.

## Supporting Information

Supporting Information S1 **Scatter plots of the correlations of MDHAQ with HAQ, HAD and SF-36.**
(DOCX)
