# Supplementary material for: Multi-Dimensional Health Assessment Questionnaire in China: Reliability, Validity and Clinical Value in Patients with Rheumatoid Arthritis
Source: PLoS One. 2014 May 21;9(5):e97952. doi: 10.1371/journal.pone.0097952 (PMC4029794; doi:10.1371/journal.pone.0097952)
Supplement: Supporting Information S1 — Scatter plots of the correlations of MDHAQ with HAQ, HAD and SF-36. (DOCX) [file pone.0097952.s001.docx]

**Scatter plots of the correlations of MDHAQ with** **HAQ, HAD and SF-36**

1. FN with HAQ, HAD and SF-36


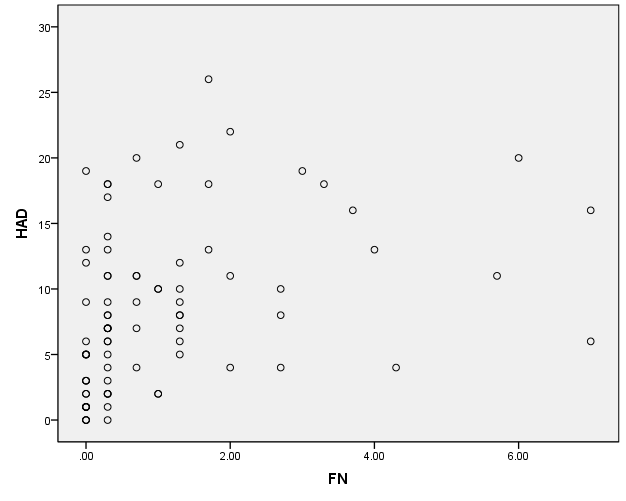

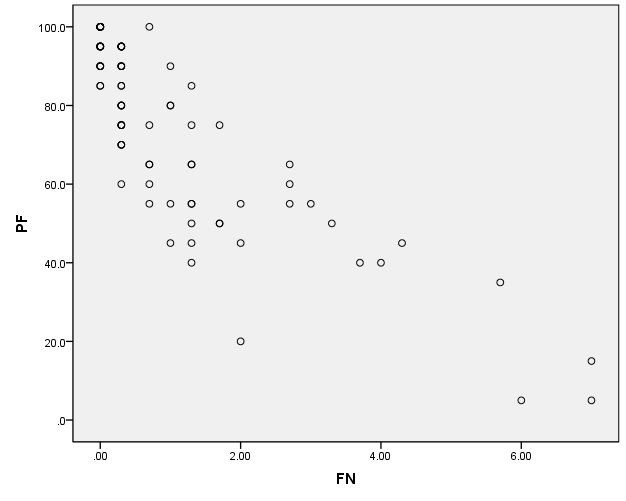


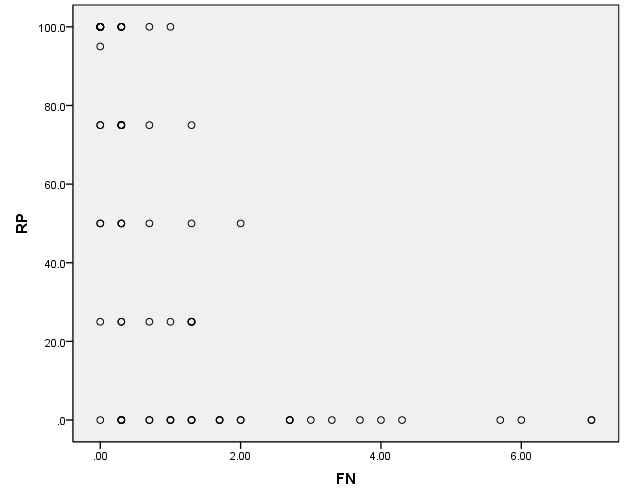

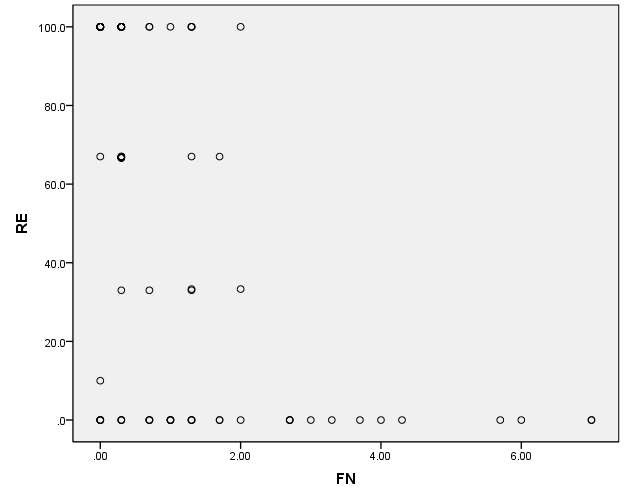


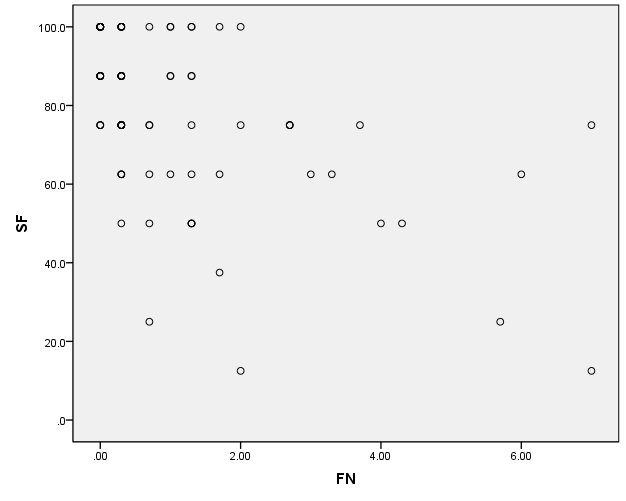

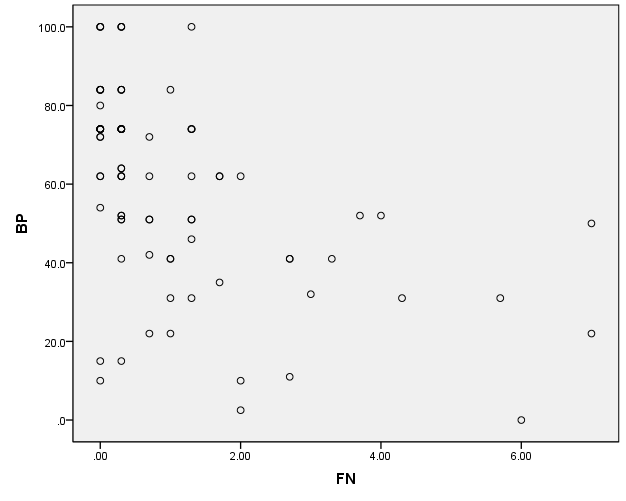


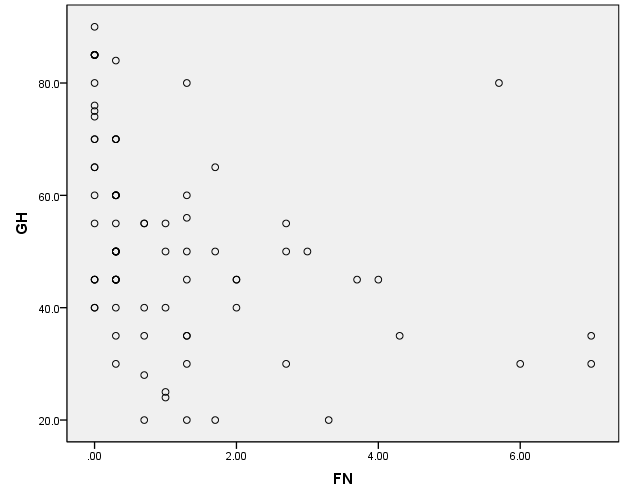

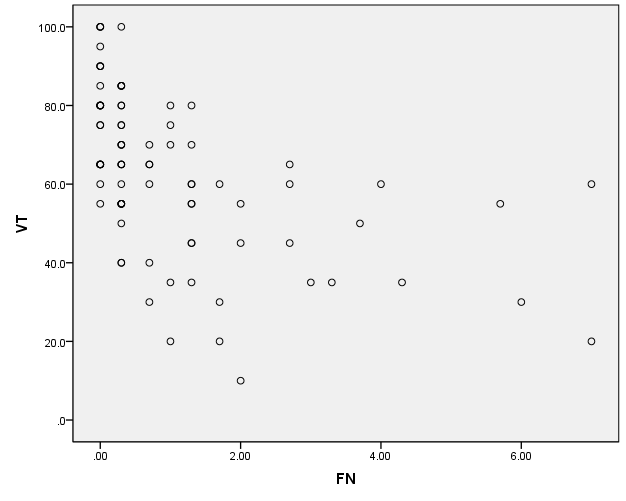


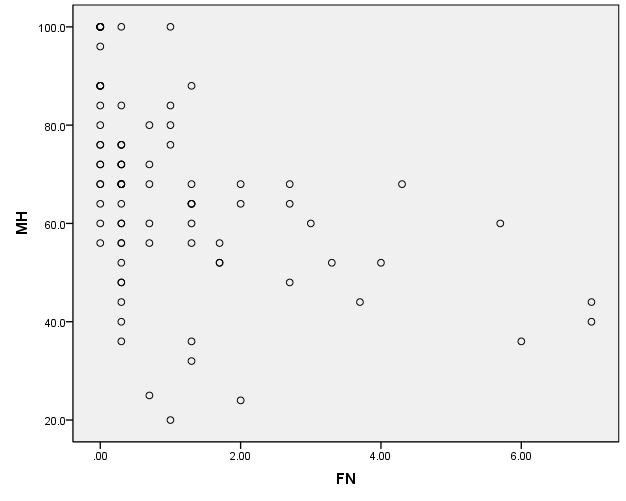


2. PS with HAQ, HAD and SF-36


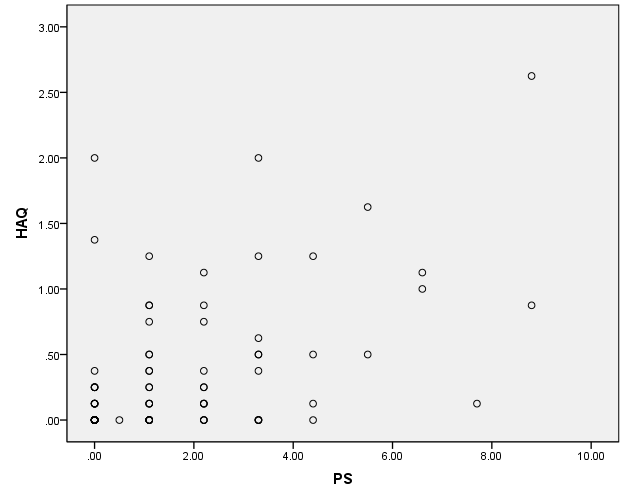

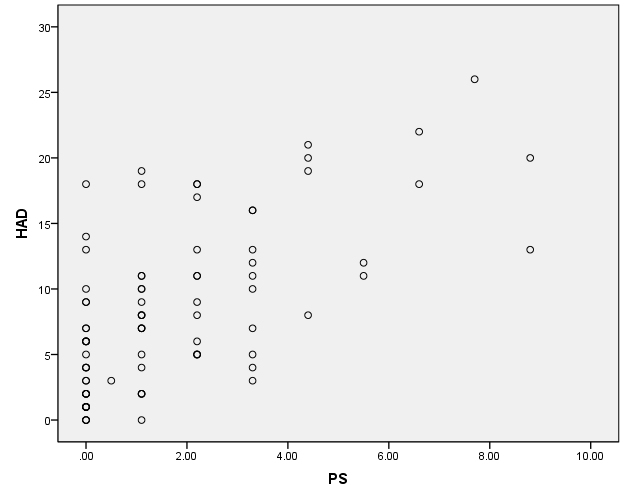


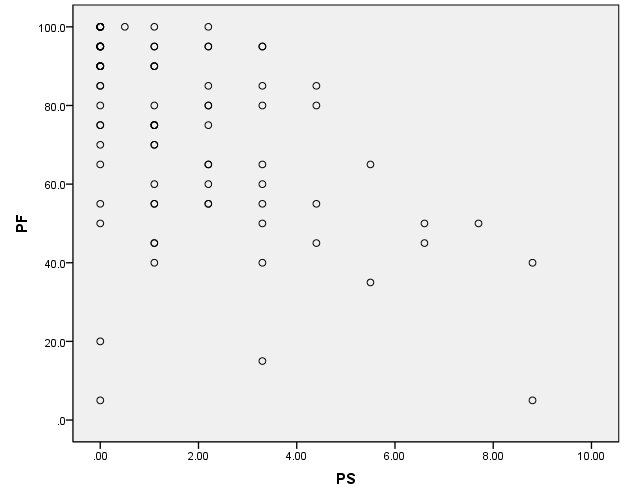

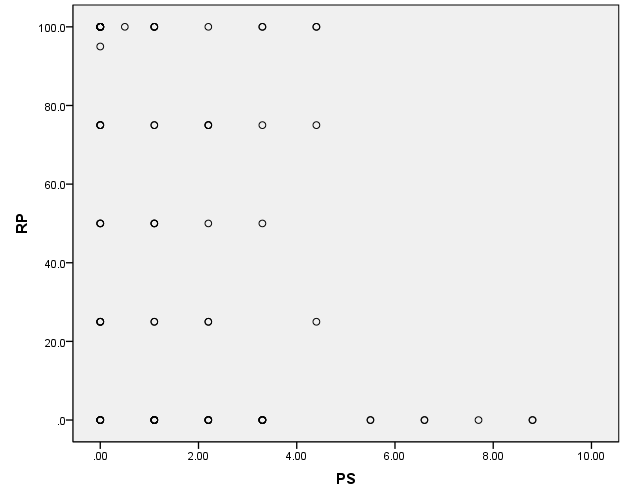


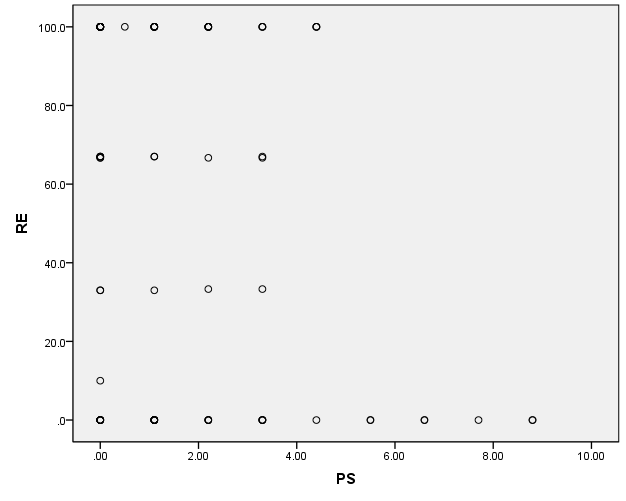

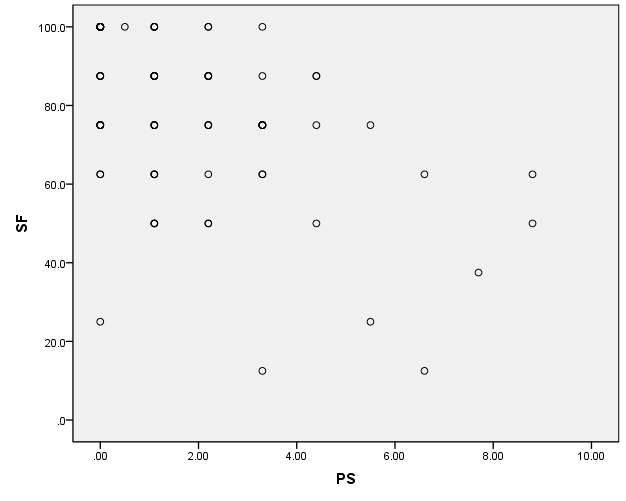


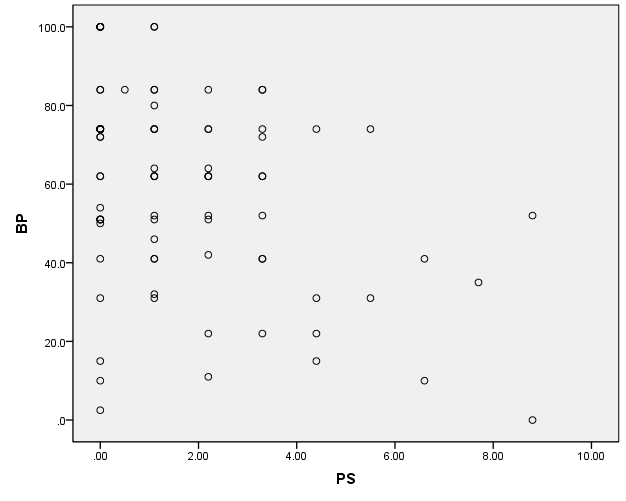

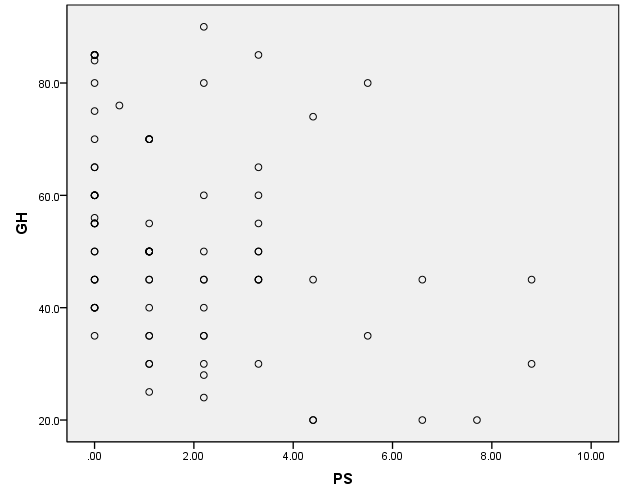


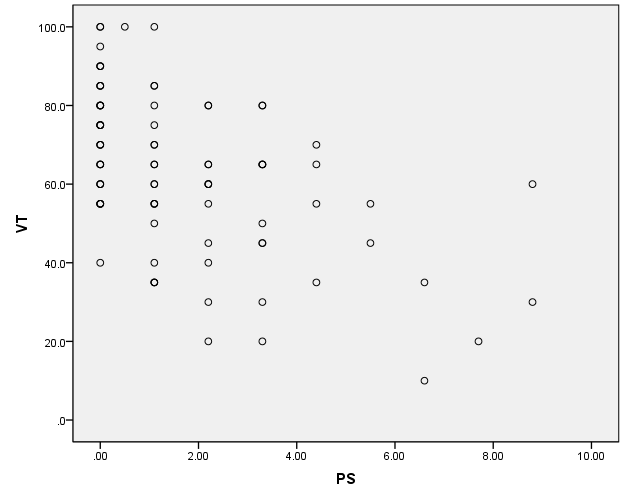

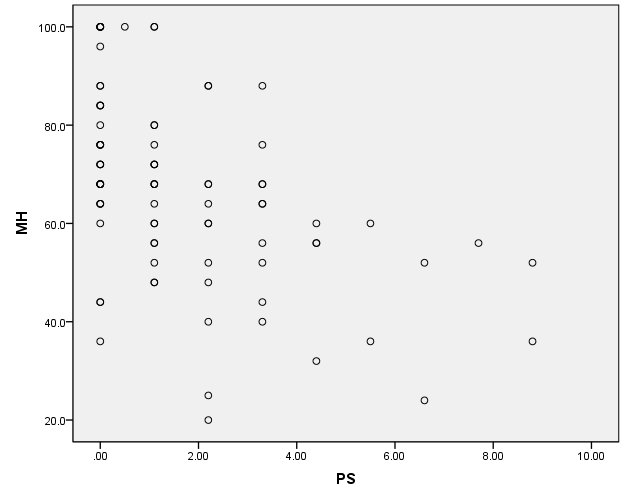


3. PN with HAQ, HAD and SF-36


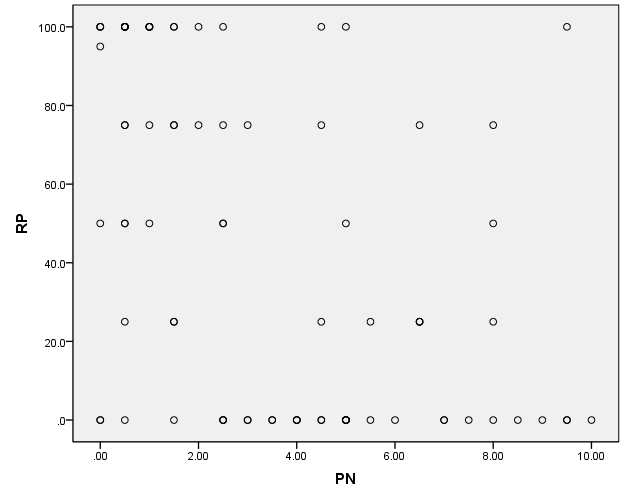


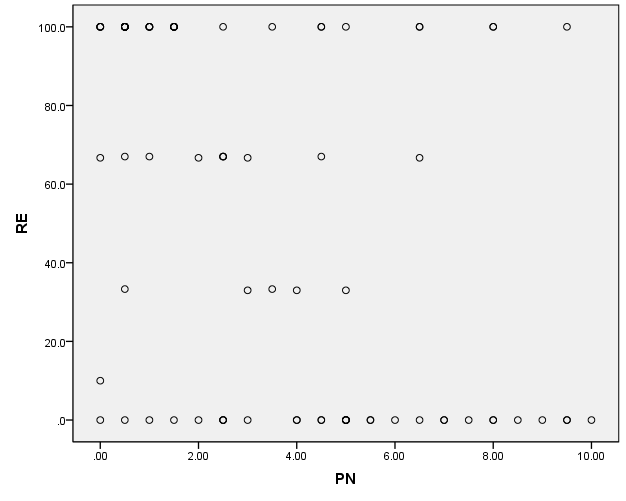

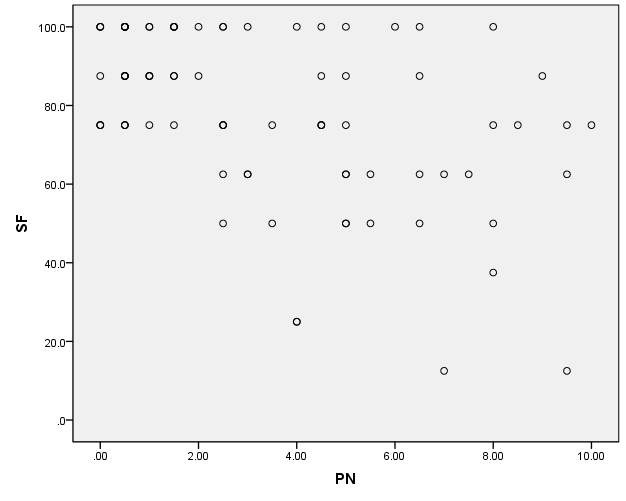


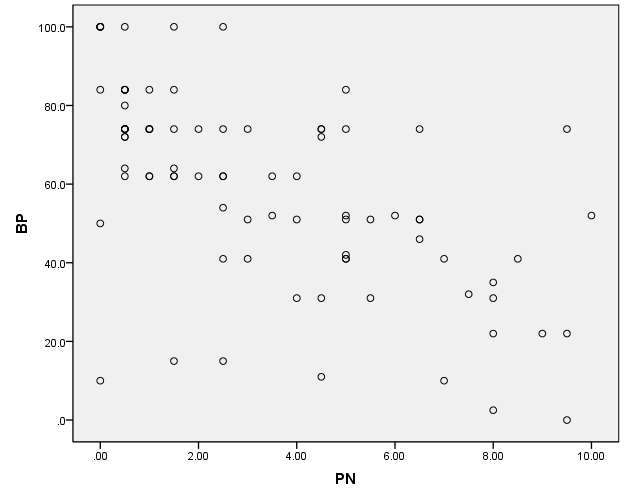

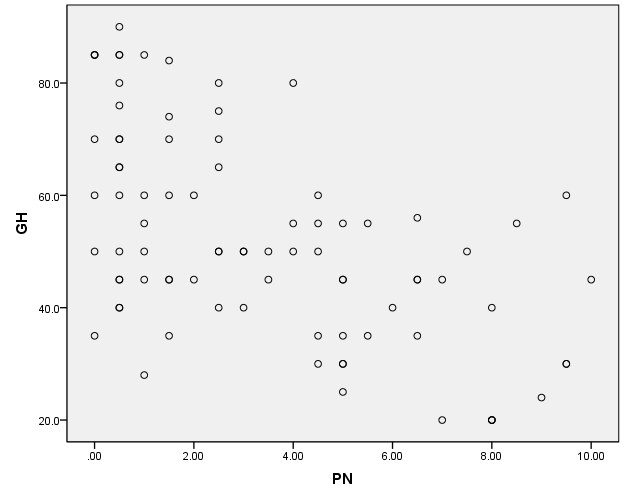


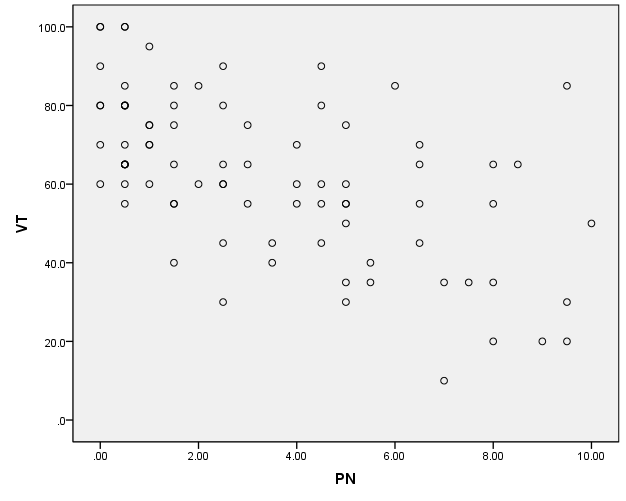

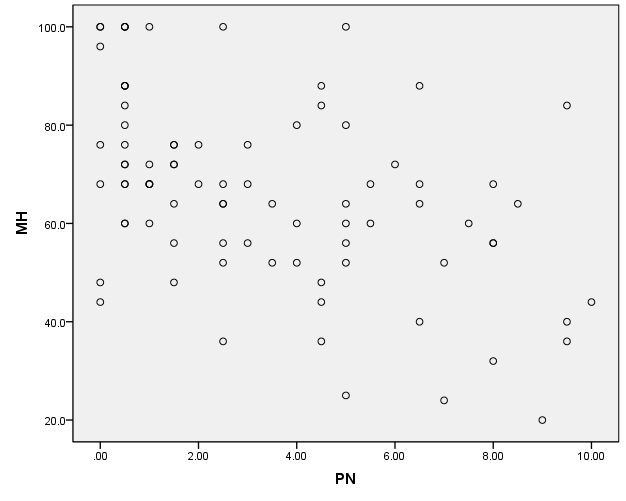


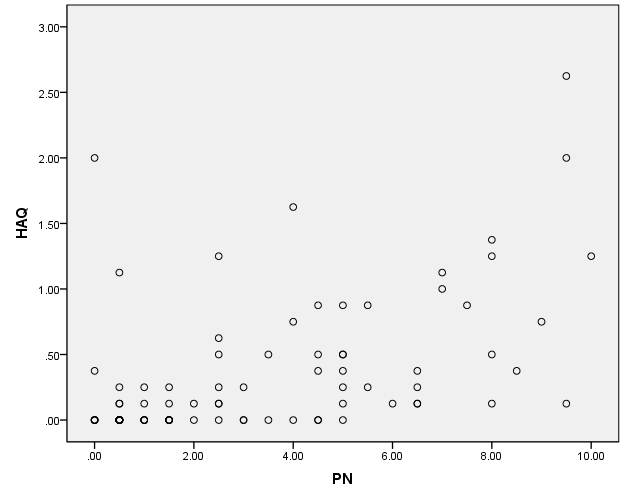

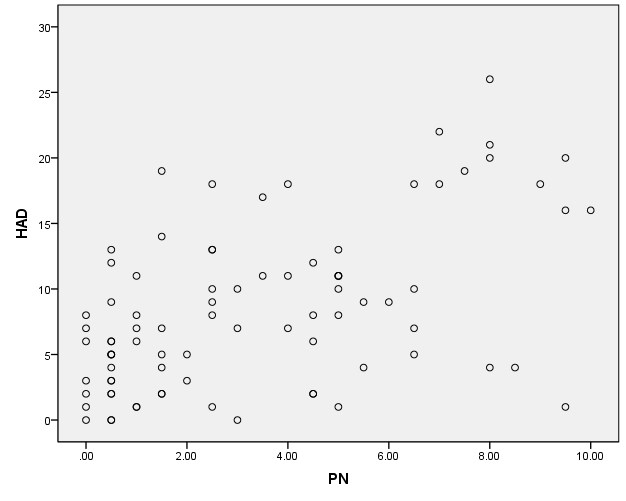


4. PTGL with HAQ, HAD and SF-36


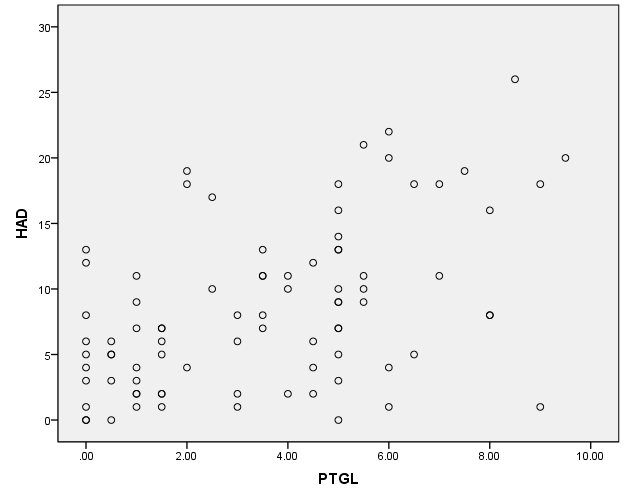

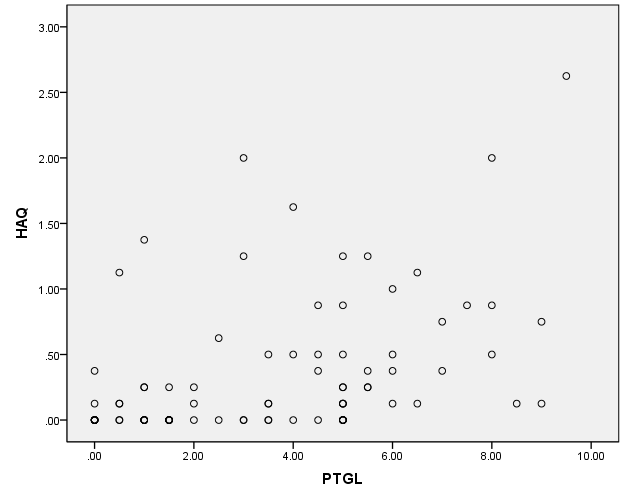


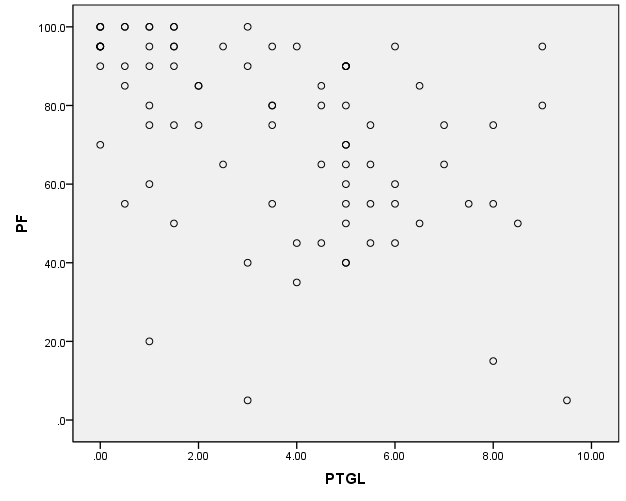

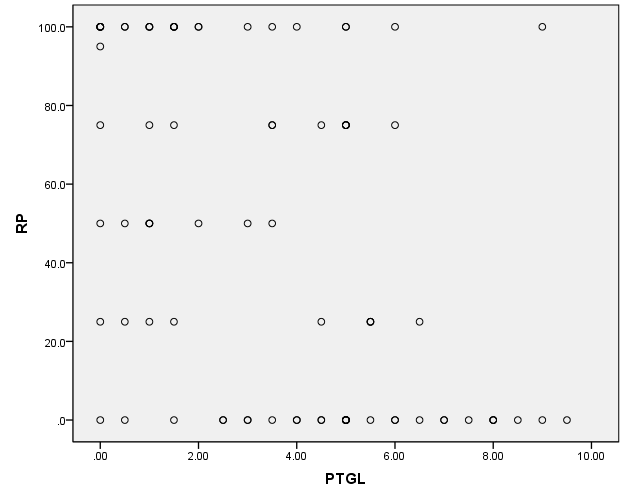


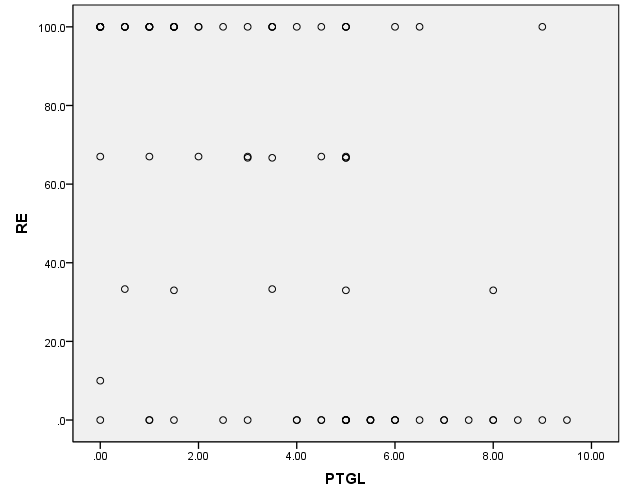

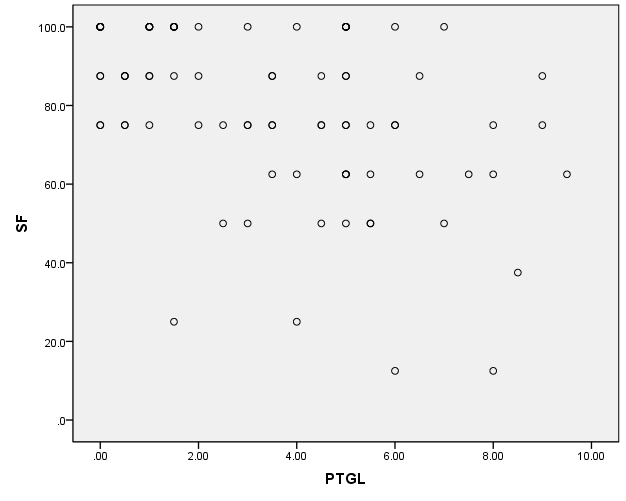


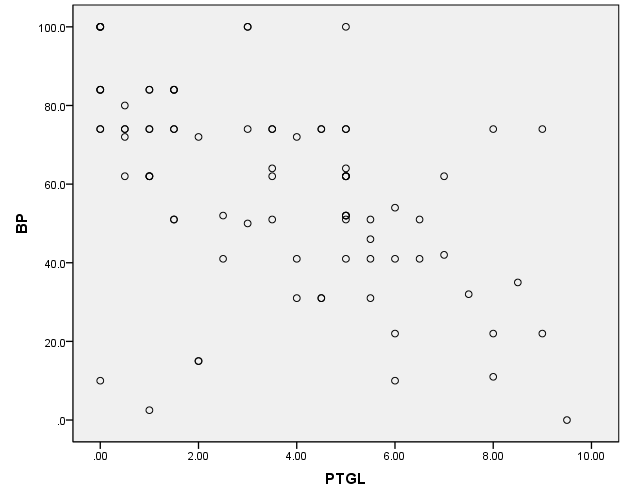

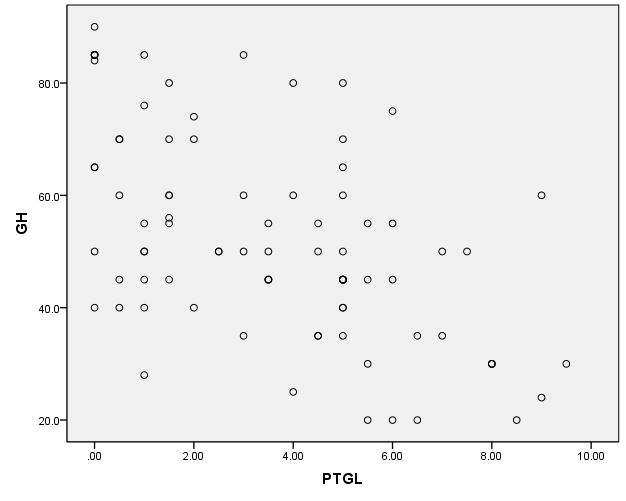


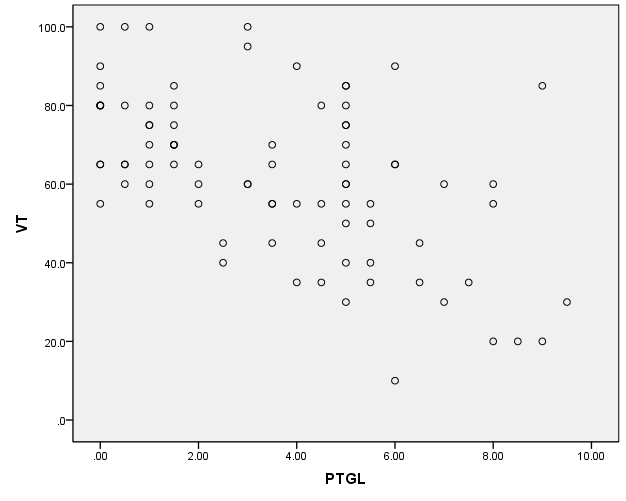

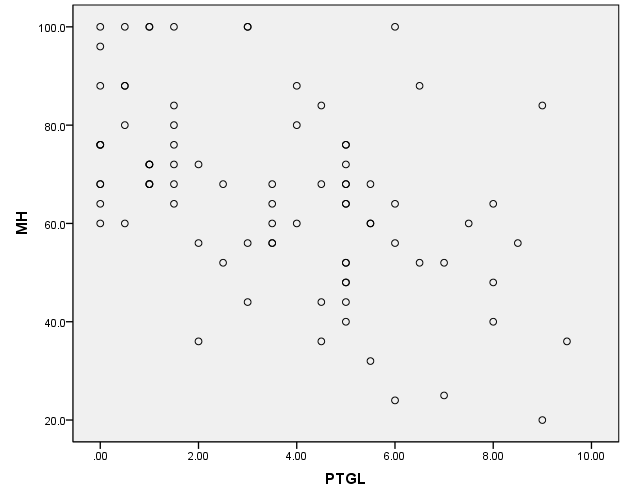


5. FT with HAQ, HAD and SF-36


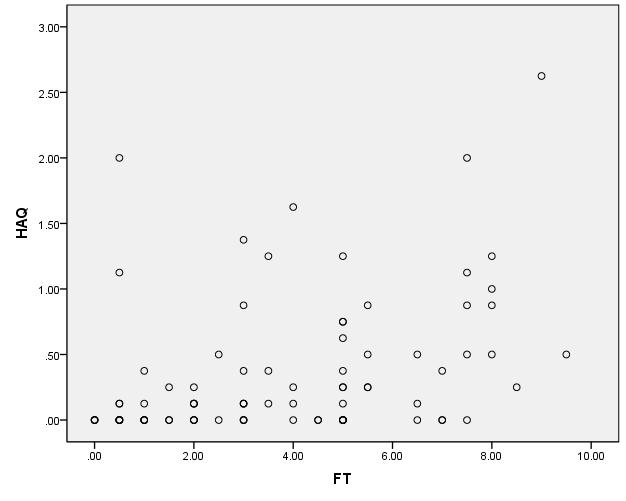

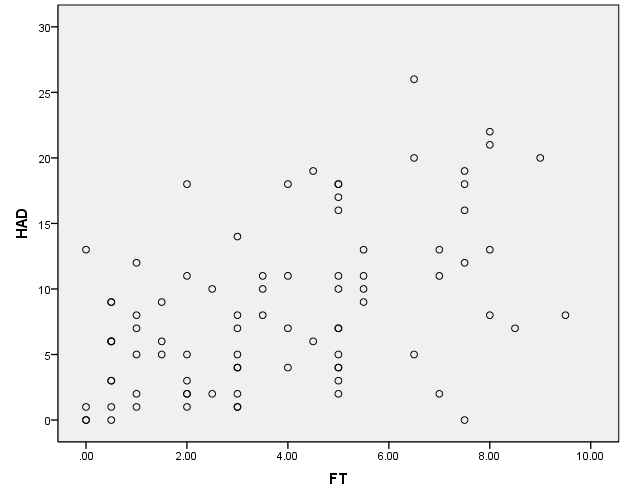


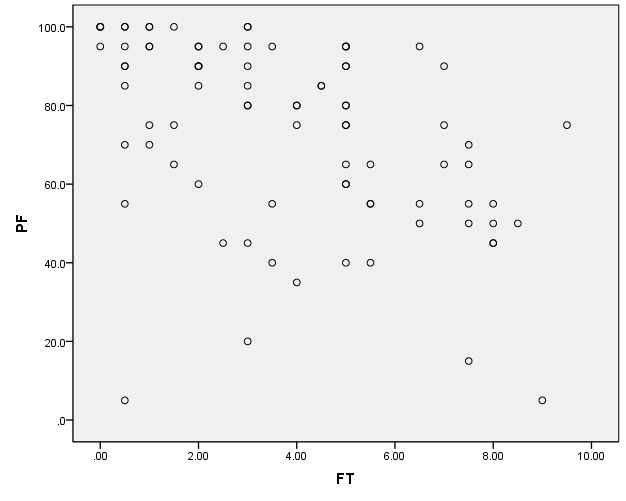

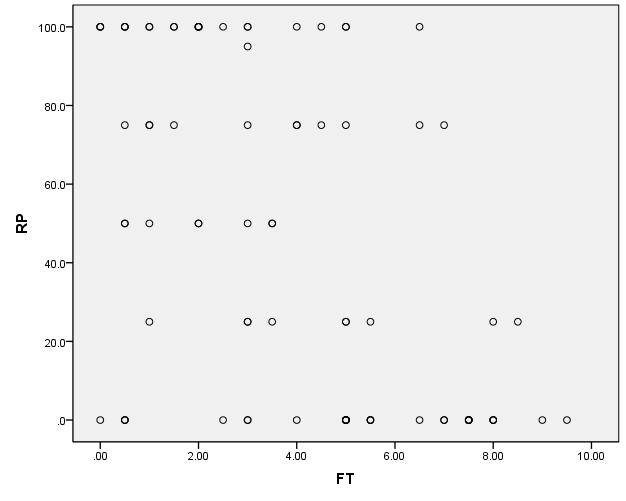


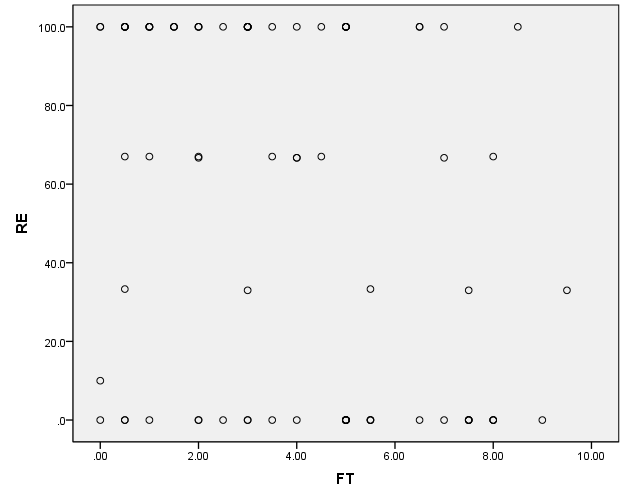

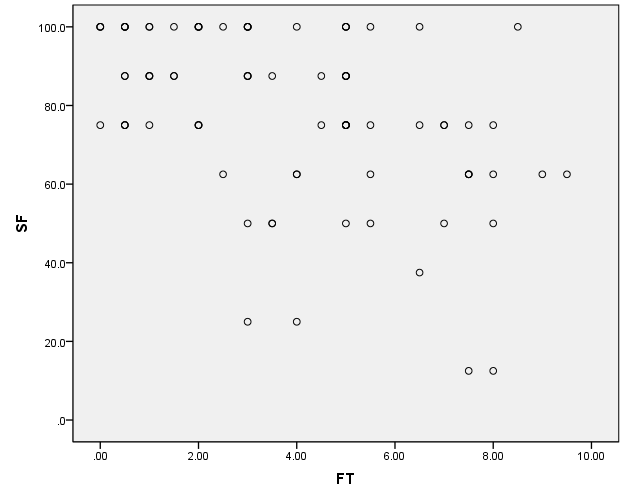


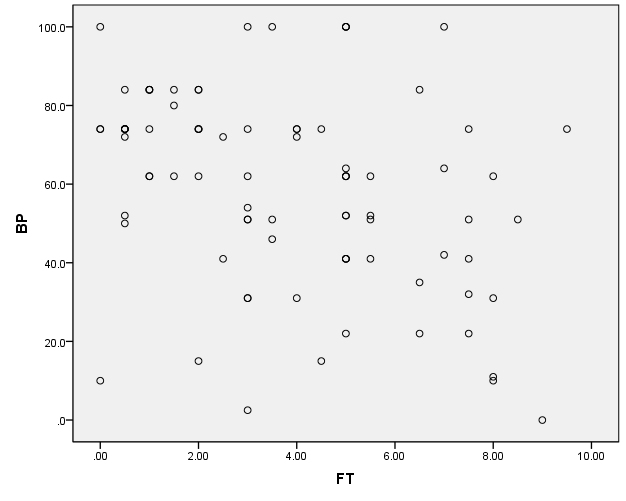

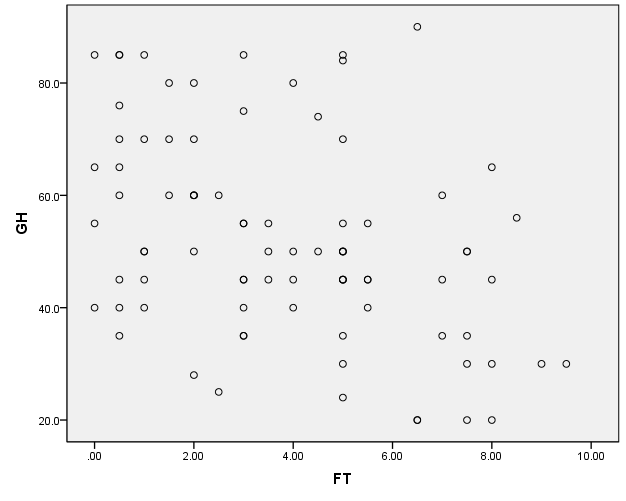


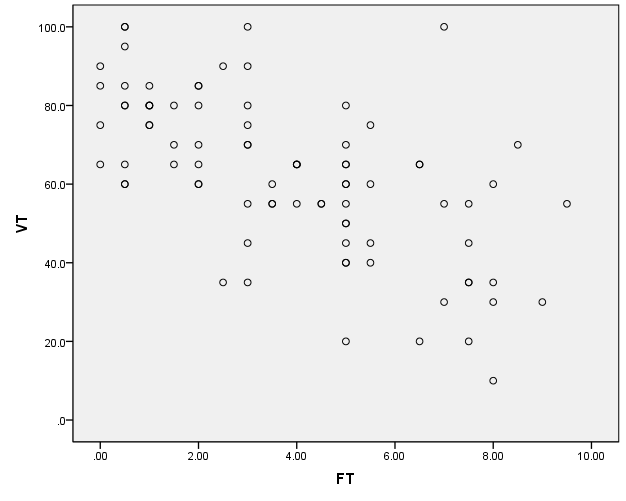

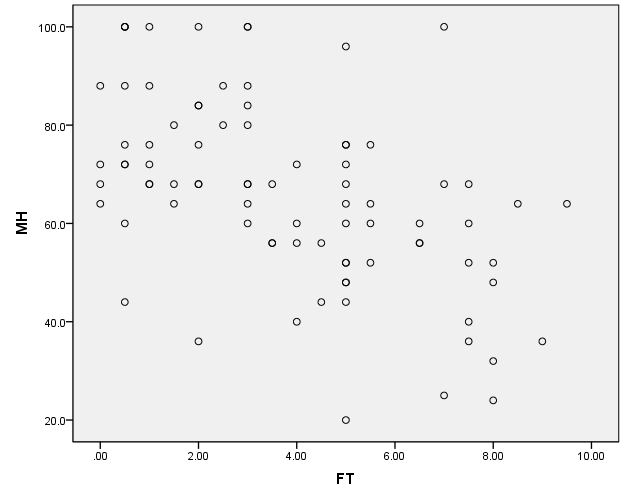


6. JCTC with HAQ, HAD and SF-36


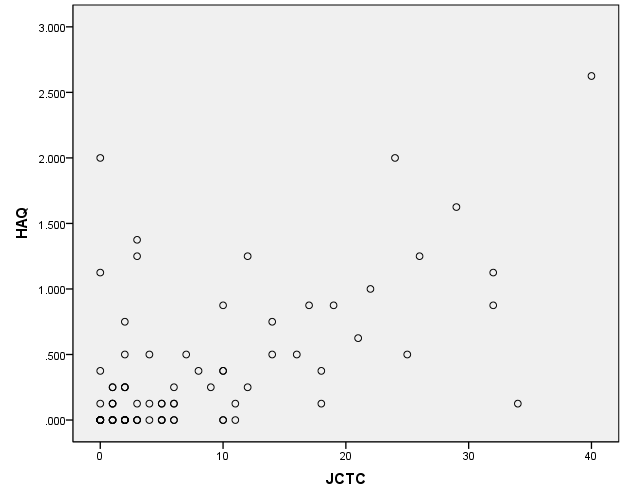

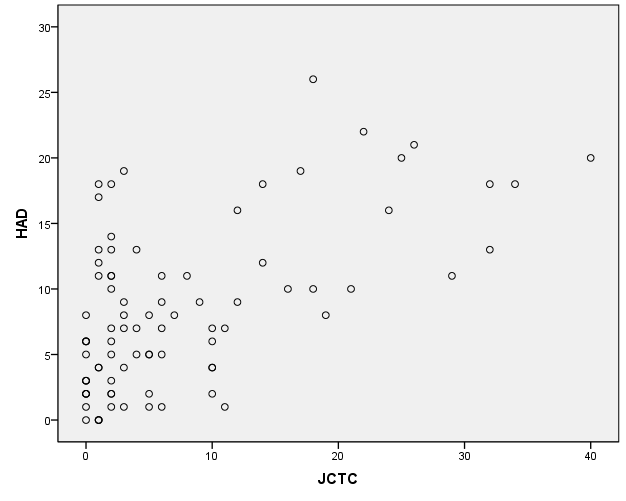


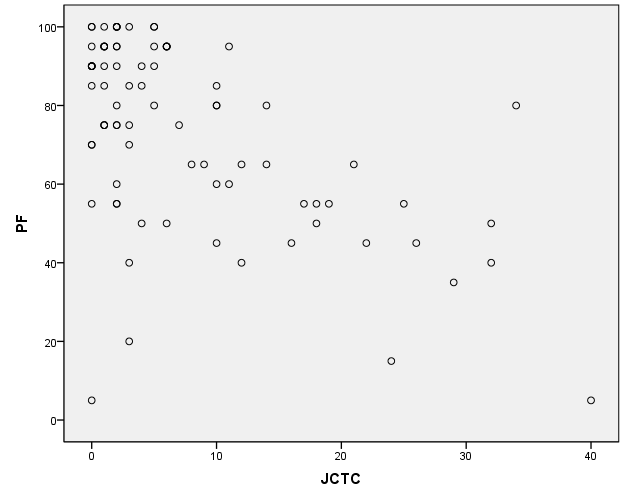

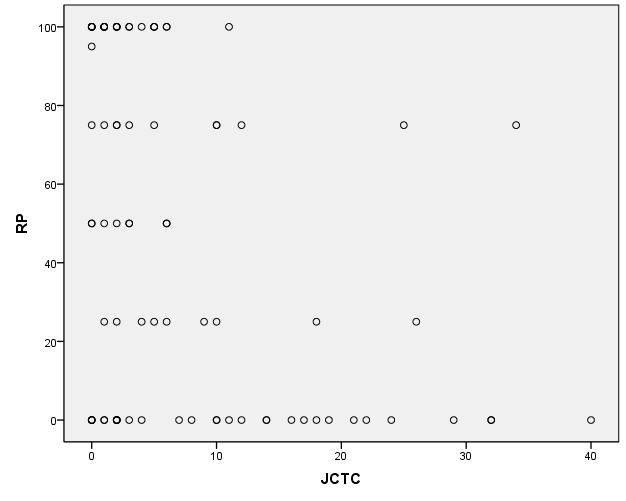


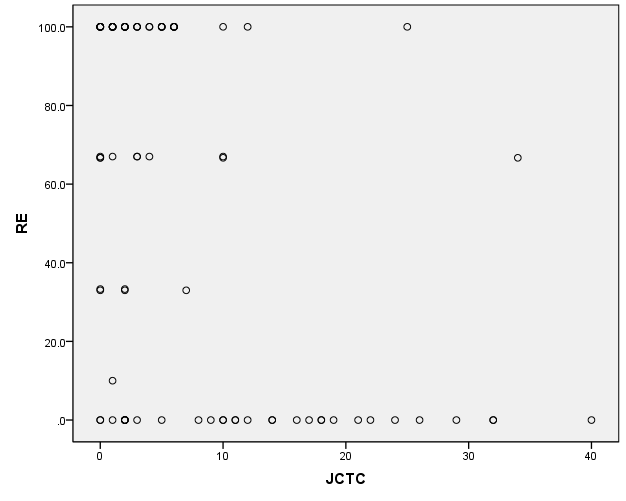

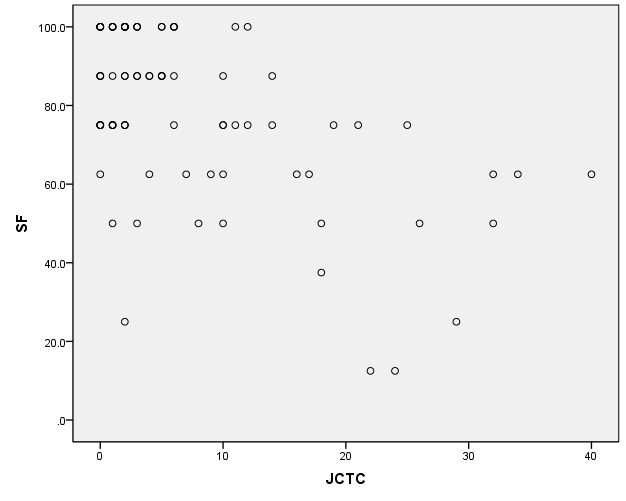


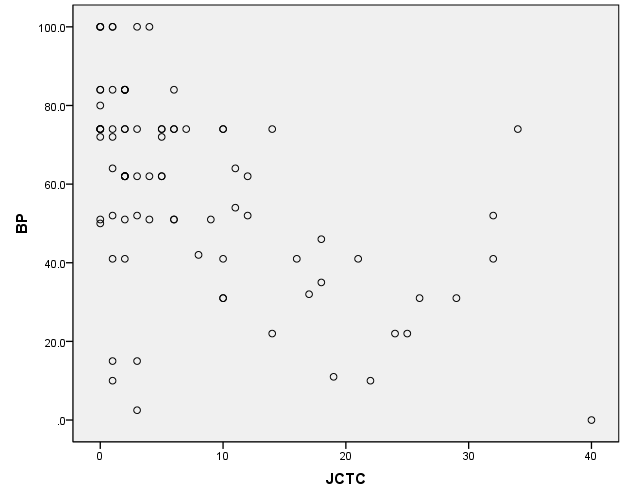

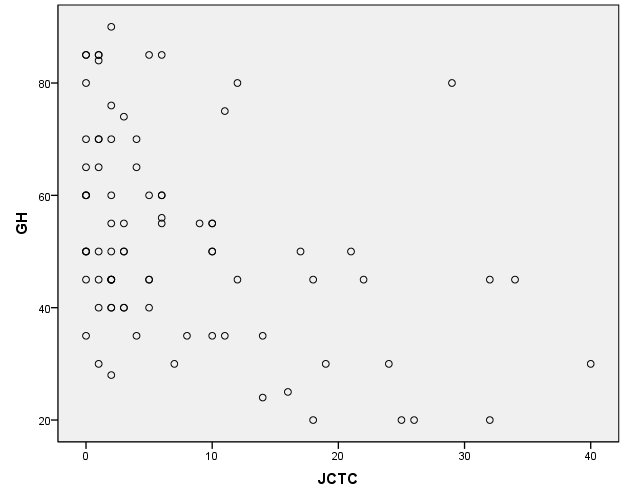


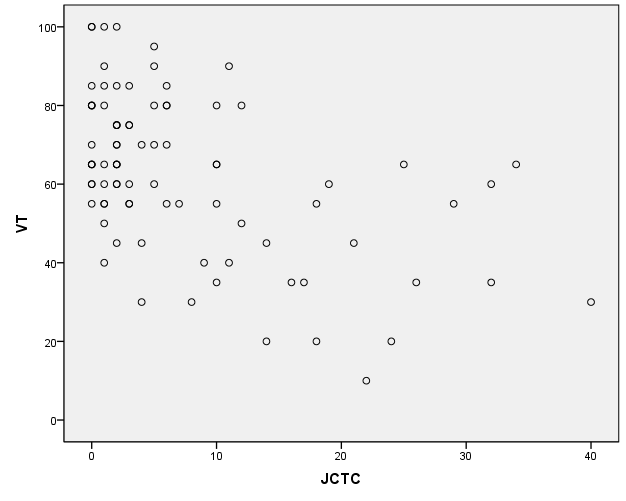

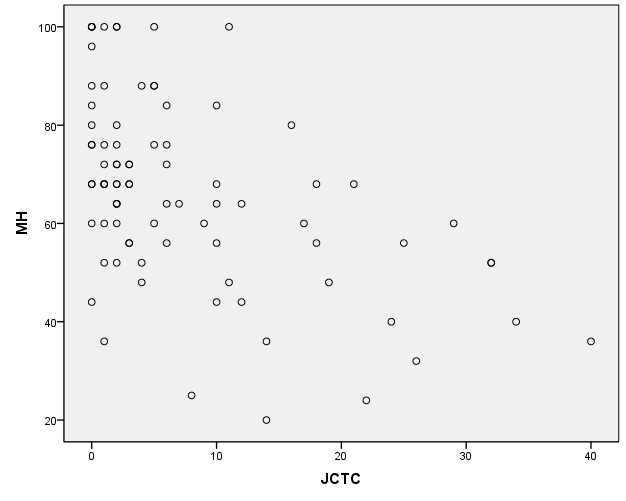


7. EX with HAQ, HAD and SF-36


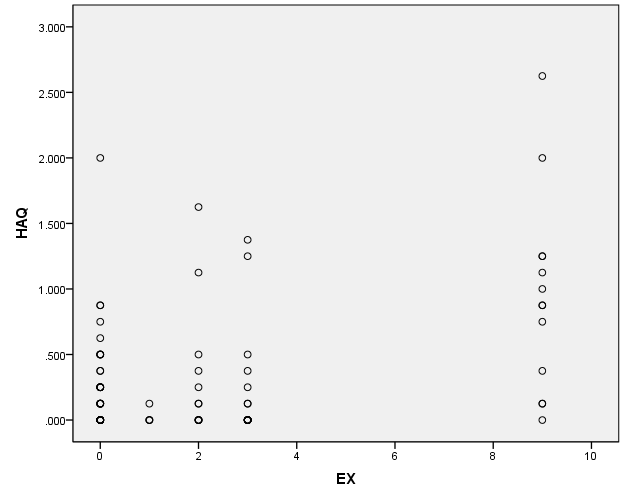

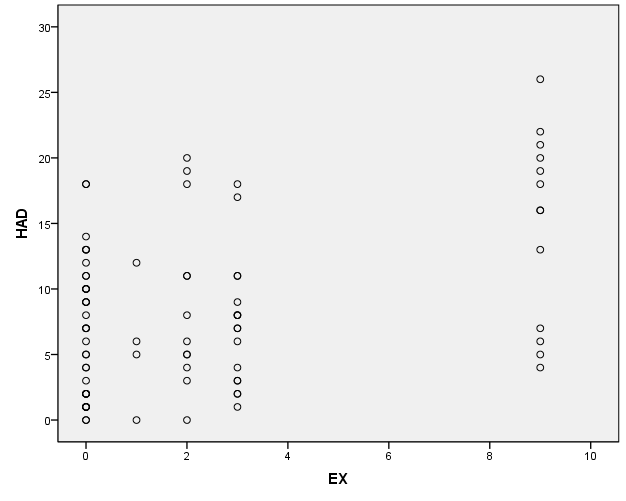


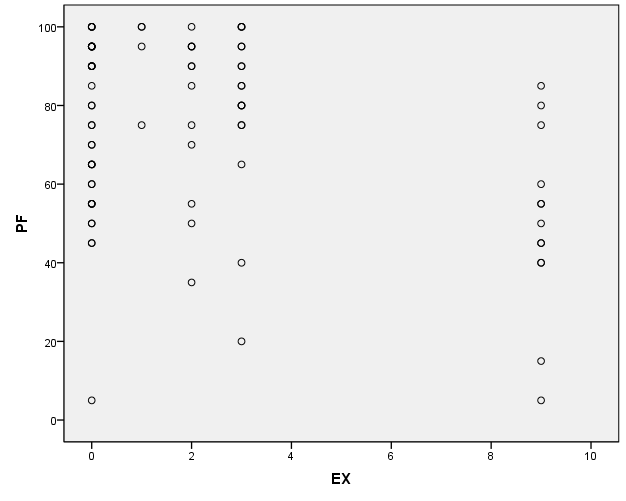

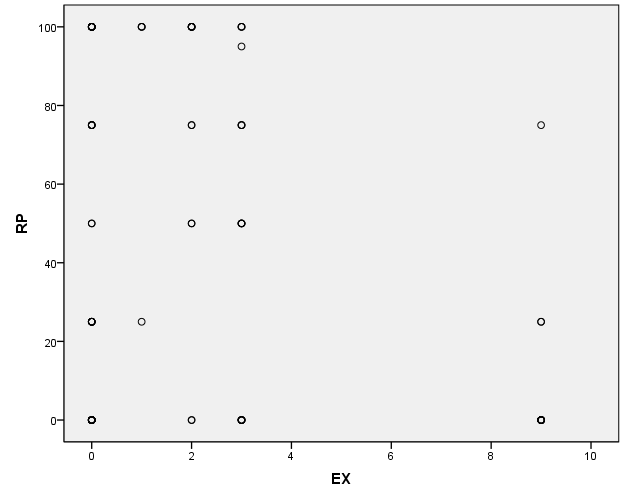


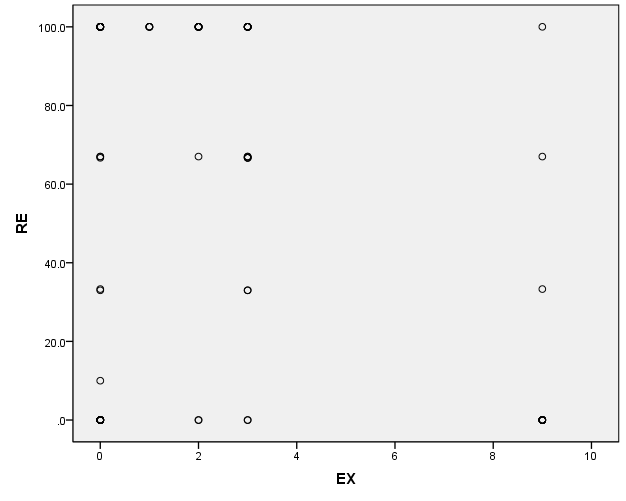

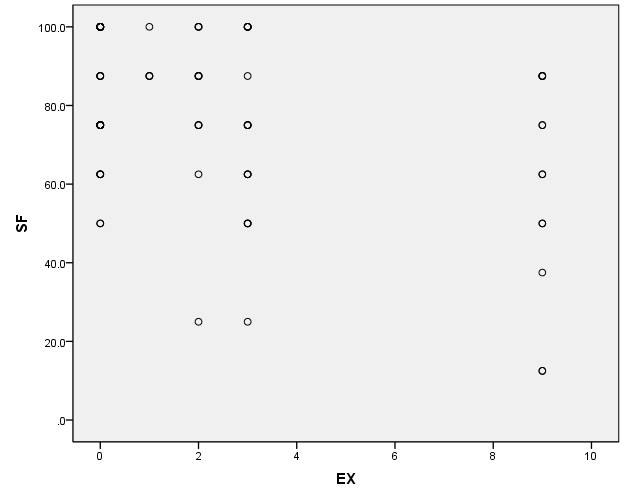


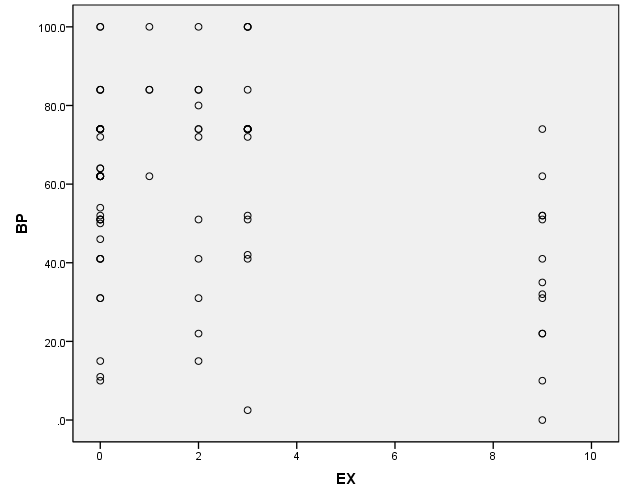

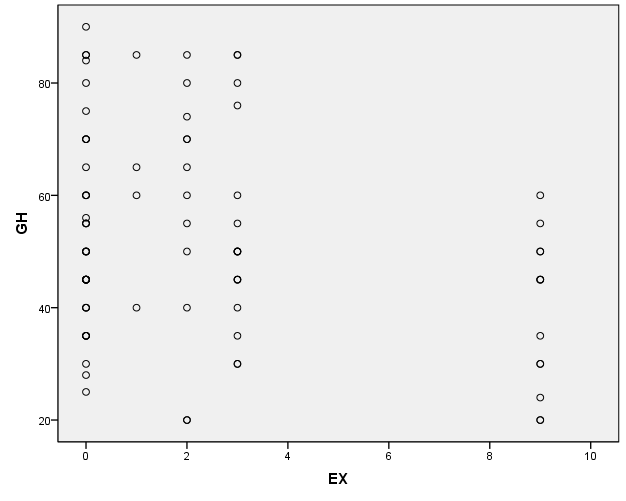


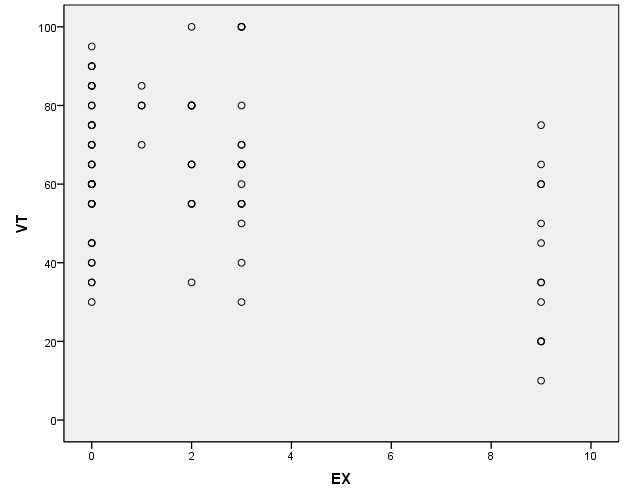

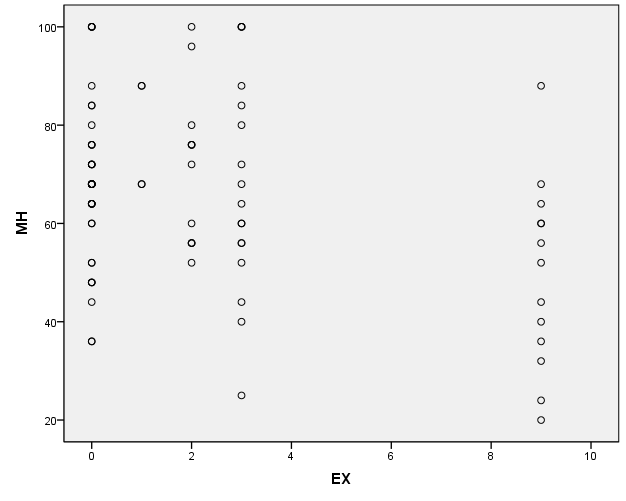


**Box-plots of RAPID3, DAS28 and CDAI**
